# The Potential Effect of Bualuang (White *Nelumbo nucifera* Gaertn.) Extract on Sperm Quality and Metabolomic Profiles in Mancozeb-Induced Oxidative Stress in Male Rats

**DOI:** 10.3390/life15010006

**Published:** 2024-12-24

**Authors:** Jiraporn Laoung-on, Pimchanok Nuchniyom, Ketsarin Intui, Churdsak Jaikang, Kanokporn Saenphet, Kongsak Boonyapranai, Giatgong Konguthaithip, Nopparuj Outaitaveep, Sasitorn Phankhieo, Paiwan Sudwan

**Affiliations:** 1 Department of Anatomy, Faculty of Medicine, Chiang Mai University, Chiang Mai 50200, Thailand; pimchanok.anatomy1@gmail.com (P.N.); ketsarin.intui@gmail.com (K.I.); sasitorn_pha@cmu.ac.th (S.P.); 2 Office of Research Administration, Chiang Mai University, Chiang Mai 50200, Thailand; 3 Research Institute for Health Sciences (RIHES), Chiang Mai University, Chiang Mai 50200, Thailand; kongsak.b@cmu.ac.th; 4 Toxicology Section, Department of Forensic Medicine, Faculty of Medicine, Chiang Mai University, Chiang Mai 50200, Thailand; churdsak.j@cmu.ac.th (C.J.); kongkiat.shang@gmail.com (G.K.); 5 Department of Biology, Faculty of Science, Chiang Mai University, Chiang Mai 50200, Thailand; kanokporn.saenphet@cmu.ac.th; 6 School of Health Sciences Research, Research Institute for Health Sciences (RIHES), Chiang Mai University, Chiang Mai 50200, Thailand; nop.outaitaveep@gmail.com

**Keywords:** environment toxicant, oxidative stress, *Nelumbo nucifera* petal, male reproductive health, ^1^H-NMR, sperm, toxicity

## Abstract

Mancozeb (MZ), an EBDC fungicide, has been found to contaminate agricultural products and cause male reproductive toxicity. The phytochemical compounds of white *N. nucifera* petal extract (WNPE) and its effects on metabolomic profiles and reproductive function in male rats poisoned with MZ were investigated. Seventy-two mature male Wistar rats were divided into nine groups (*n* = 8) and, for 30 days, were gavaged with WNPE at doses of 0.55, 1.10, and 2.20 mg/kg; were given distilled water; or were co-gavaged with MZ and WNPE. By evaluating the ^1^H-NMR of WNPE, myricetin, apigenin, luteolin, ferulic acid, caffeic acid, ascorbic acid, genistein, chlorogenic acid, naringenin, and ellagic acid were found, and the essential minerals were evaluated by AAS. The NMR spectra demonstrated that creatine, carnitine, ACh, and choline in WNPE were significantly higher than that in MZ. The gavaging of the rats with WNPE before poisoning them with MZ improved creatine, carnitine, acetylcholine, progressive sperm motility, sperm viability, and normal sperm morphology compared to rats who only received MZ. It was concluded that MZ had a toxicity effect on the male reproductive system via decreased metabolomic profiles, affecting sperm motility, sperm viability, and normal sperm morphology. Nevertheless, WNPE had plenty of bioactive compounds that could enhance creatine, carnitine, and acetylcholine, which are related to sperm quality in male rats. WNPE should be considered as an alternative dietary supplement that can protect against MZ toxicity and enhance sperm quality in the male rat reproductive system.

## 1. Introduction

Mancozeb (MZ), a fungicide belonging to the ethylene bis dithiocarbamate (EBDC) family, is extensively utilized for the management of fungal infections in agricultural and industrial situations [1,2]. MZ has been shown to contaminate agricultural produce and the environment, leading to oxidative stress and other detrimental effects on both animals and humans [2,3]. A previous study found that chronic exposure to a metabolite of EBDCs resulted in adverse effects, including through significant dermal exposure to MZ in agricultural workers and the general population consuming products with pesticide contamination [4]. Chronic exposure to MZ can cause increased urinary excretion of manganese and ETU, hemoglobin-ETU adducts, and altered thyroid function [4]. Moreover, several studies found that MZ had a toxicity effect in the thyroid [5], liver [6], kidney [7], nervous system [8], gastrointestinal (GI) tract [4], and reproductive organs [9]. Additionally, MZ-induced lipid peroxidation markers [6], while reducing serotonin and picolinic acid levels [8], suggested that MZ causes increased oxidative stress and toxicity in rats. It has been found to induce oxidative stress and result in testicular dysfunction, epididymal abnormalities, and spermatozoa anomalies [10,11]. Thailand is an agricultural country, with over half of its inhabitants employed in agriculture [12]. Recently, Thailand’s population growth significantly decreased [12]. It is possible that the male population in Thailand is exposed to MZ toxicity for a considerable and prolonged period, which may affect the reproductive system and population growth in Thailand. Preventing and managing oxidative stress can benefit male reproductive health.

Male reproductive dysfunction can be caused by lifestyle choices, particular vocations, age-related disorders, and environmental toxicant exposure, all of which result in the production of oxidative stress [13]. Oxidative stress induces cellular damage [14,15], reducing sperm production and male fertility [16,17,18]. It could change the metabolomic profile, affecting creatine, carnitine, acetylcholine, choline, and xanthurenic acid levels, which are related to male reproductive function [19]. Conventional serum profile testing procedures are established laboratory techniques used to examine samples for numerous biochemical indicators. Spectrophotometry, enzymatic tests, immunoassays, chromatography, and electrolyte analyzers are common testing techniques used [20,21,22]. However, traditional serum profiling approaches employ a variety of methodologies to precisely assess numerous biochemical indicators in serum, which is time-consuming, sample-intensive, and costly. NMR metabolomics is currently a technique that uses fewer samples for examination and allows for the quick and repeatable collection of metabolite profiles [23].

Conventional oxidative stress prevention and management involves antioxidant consumption [13], such as consuming vitamin E (Vit E), which is one of the most commonly consumed antioxidants for preventing and managing MZ toxicity [24]. Artificial antioxidants are more effective and more accessible to administer, but they are more costly and have serious adverse effects such as DNA synthesis disruption, carcinogenesis, fibrosis, and embryotoxicity [25,26]. Therefore, natural antioxidants are essential. Plants rich in phytochemical substances possess a high concentration of natural antioxidants and can be used as an alternate source of oxidative defenses in male reproductive dysfunction [13,16] and may be developed and used to decrease MZ toxicity.

Polyphenols are a group of chemical substances that are common in plants and have benefits including scavenging free radicals, enhanced sperm concentration, and sperm motility [27]. Plants or herbal remedies are traditionally and widely used against oxidative stress and enhanced male reproductive function in animals, such as *B. rotunda* rhizomes [28], *K. parviflora* rhizomes [29], *M. oleifera* leaves [30], *Z. officinale* [31], and *N. nucifera* petal [11,32].

*Nelumbo nucifera* Gaertn. (*N. nucifera*), or “Bualuang”, belongs to the Nelumbonaceae family [33]. It is used as a food and tea, including in traditional medicine [34,35,36], and notably the reddish-pink stamens of *N. nucifera* have been mentioned in the National List of Essential Medicines and are a well-known herbal remedy for heart nourishment in Thailand [37], but the petals are not used and become agri-food waste and by-products of agricultural, herbal medicine, and food industry processes. In an in vitro study, the white *N. nucifera* petal extract was found to have phytochemical contents and an antioxidant potential higher than those of the red petals [32]. Moreover, it has been reported that *N. nucifera* seeds contain iron, manganese, zinc, copper, magnesium, calcium, potassium, chromium, and sodium [34], of which magnesium, zinc, and calcium play essential roles in sperm motility [38]. However, the minerals of the *N. nucifera* flower have received little scientific attention. A phytochemical study of white *N. nucifera* petals using liquid chromatography–mass spectrometry (LC-MS) showed they contained (+)-delta-tocopherol; kaempferitrin; ouabain; convallatoxin salasodsalasodine; isorhamnetin-3-O-rutinoside; 20, 3, 3′4, 40-pentahydroxy-4′-glucosulchalcone; 4,8′-Bi((+)-epicatechin); and quercetin-3-O-arabinoglycoside [11]. However, the specific bioactive compounds in plant extracts can be evaluated by high-quality tools such as NMR and atomic absorption spectroscopy (AAS) for the detailed examination of phytochemicals. Our previous study revealed that white *N. nucifera* petals improved cattle sperm quality after MZ exposure in an in vitro model [11]. Although white *N. nucifera* petals are rich in phytochemicals and affect male reproductive function in an in vitro model, its effect on male reproductive function and metabolic profiles in an in vivo model induced with MZ has not yet been investigated.

Therefore, the current study proposes to examine the protective effects of white *Nelumbo nucifera* petal extract (WNPE) on metabolomic profiles and male reproductive function in Wistar rats exposed to Mancozeb (MZ)-induced oxidative stress. Specifically, the study aimed to identify the phytochemical and mineral composition of WNPE through 1H-NMR and atomic absorption spectroscopy (AAS), assess its impact on key metabolites such as creatine, carnitine, acetylcholine, and choline, and evaluate its potential to improve sperm motility, viability, and morphology in comparison to MZ-treated and control groups. By exploring WNPE’s antioxidant properties and its role in mitigating MZ toxicity, the study sought to determine whether WNPE could serve as a natural dietary supplement for protecting male reproductive health against environmental toxicants.

## 2. Materials and Methods

### 2.1. Chemical Preparation

Commercial MZ (80% WP) was purchased from Sotus International Company Ltd. (Nonthaburi, Thailand); its primary metabolite is ethylene thiourea (ETU). Commercial MZ was used in the experiment due to its familiarity in normal human use and contamination. MZ was dissolved in olive oil at a dose of 500 mg/kg for the experiment. Olive oil was used as the vehicle control.

### 2.2. Plant Preparation and Extraction

The fully grown petals of Bualuang were collected from the Thung Yang subdistrict, Laplae district, Uttaradit Province, Thailand (17°31009.7″ N, 99°59001.6″ E). The specimen was deposited and authenticated at the Herbarium, Faculty of Pharmacy, Chiang Mai University (voucher specimen number is 023248–2). The petals were washed, steamed, and dried at 60 °C. The dried white petals were extracted with hot distilled water for five minutes (white *N. nucifera* petals extract (WNPE)), and the extract was dried by lyophilization with a 12.5% yield and stored at −20 °C before experimentation [6,8,11].

### 2.3. Phytochemical Screening of WNPE Using Proton Nuclear Magnetic Resonance (^1^H-NMR)

The dried WNPE crude extract obtained through lyophilization was screened for the phytochemical contents by NMR analysis at the Central Science Laboratory, Faculty of Science, Chiang Mai College, using a nuclear magnetic resonance spectrometer (Bruker NEO 500 MHz, Bruker Daltonics, Billerica, BA, USA) according to a previous study [6]. Each chemical compound was identified by using the library and each peak of every non-targeted metabolite needed to be identified and adjusted by less than 0.01 when compared to that from the library.

### 2.4. Mineral Assay with Atomic Absorption Spectroscopy (AAS)

Dried WNPE (10 mg) was prepared with ultrapure deionized water and preserved at 4 °C for analysis. Mg, Zn, and Ca concentrations in previously mineralized samples were determined using Agilent 240FS AA (200 series AA) (Santa Clara, CA, USA). The elements were examined using atomic absorption. Instruments were calibrated to recognized standards. Calibration curves were used to quantify minerals.

### 2.5. Animals

Seventy-two mature male *Wistar* rats aged 6–8 weeks with weights of 220–240 g were purchased from Nomura Siam International CO., Ltd., Bangkok, Thailand, and the experimental protocol was approved by the Animal Ethics Committee, Faculty of Medicine, Chiang Mai University (No. 6/2564). The animals were acclimatized under standard laboratory conditions for at least one week before the experiment at the Animal Laboratory Building, Faculty of Medicine, Chiang Mai University, with a controlled temperature of 25 ± 2 °C, a 12 h dark/12 h light cycle, and access to a standard pelleted diet and filtered water.

### 2.6. Experimental Design

The seventy-two animals were randomly divided into 9 groups (*n* = 8). The nine groups were gavaged daily as follows:

– Group I (control group): male rats received 1 mL of distilled water.
– Group II–IV: male rats were orally gavaged with WNPE that was dissolved in distilled water at doses of 0.55, 1.10, and 2.20 mg/kg, respectively.
– Group V (vehicle control): male rats were orally gavaged with olive oil.
– Group VI (toxic group): male rats were orally gavaged with MZ 500 mg/kg, which is the toxicity dose that was recommended in a previous study [39].
– Group VII–IX (treatment group): male rats were orally co-gavaged with WNPE at doses of 0.55, 1.10, and 2.20 mg/kg, respectively, and with MZ.

The dose of WNPE used in this study was chosen to be similar to that in human consumption, as 1.10 mg/kg corresponds to the daily tea consumption in humans [6]. All groups were continually treated for 30 days, which is the effect duration for the treatment of MZ and plant extracts on the male rat reproductive system [39,40]. Then, all rats were anesthetized using Isoflurane and sacrificed by cardiac puncture, and blood samples were collected. The epididymis was removed, trimmed, and weighed before sperm quality studies were carried out.

### 2.7. Metabolomic Activity Analysis by ^1^H-NMR

The blood samples were analyzed using NMR analysis to determine the metabolomic profile according to a previous study [6]. Chemical shifts were referenced based on the TSP resonance (δ = 0.0), and baseline correction was performed manually. Each chemical compound was identified by using the Human Metabolome Database (HMDB), and each peak of every non-targeted metabolite needed to be identified and adjusted by less than 0.01 when compared to that of the HMDB. The quantitative resonance peaks of each molecular marker were δ = 3.02 for creatine, δ = 3.21 for carnitine, δ = 4.05 for choline, and δ = 6.80 for xanthurenic acid [41,42].

### 2.8. Sperm Quality Investigation

The sperm suspension was prepared by mincing and homogenizing the right caudal epididymis in 10 mL of Krebs media. The sperm quality study was immediately evaluated.

#### 2.8.1. Sperm Concentration and Sperm Motility Assay

The sperm concentration was determined by mixing 10 µL of sperm suspension and 10 µL of 0.4% trypan blue solution, and then 10 µL of mixture solution was dropped into the Neubauer hemocytometer. The number of duplicated sperm was determined in each rat. The sperm concentration was calculated and expressed as million/mL [28].

The sperm motility assay was modified from the conventional technique carried out in a rat sperm motility study [43]. From each rat, 20 µL of the sperm suspension was collected. The Neubauer hemocytometer was used for evaluating sperm motility at 400× magnification under a light microscope (Olympus CH2), and a total of 200 sperm were counted and classified for each rat [11,43].

#### 2.8.2. Sperm Viability and Acrosome Integrity Assay

A total of 20 µL of sperm suspension from each rat was mixed with 20 µL of trypan blue. Then, 10 µL of the mixture was added on the slide and smeared. We air-dried, fixed, and stained the slides with Giemsa solution in accordance with our previous study [11]. The sperm viability and acrosome integrity were investigated under a 1000× light microscope, and a total of 200 sperm were counted and classified for each rat.

#### 2.8.3. Sperm Morphology Assay

The TB/Giemsa-stained spermatozoa slide was used to determine the morphology under the light microscope at 1000× magnification, and a total of 200 sperm were counted and classified for each rat. Four morphological patterns of sperm were identified, which is consistent with previous reports [11,43].

### 2.9. Correlation Analysis of Metabolomic Profiles and Sperm Quality

To understand the relationship between the metabolomic profiles (creatine, carnitine, choline, and xanthurenic acid) and sperm quality, correlation analyses were carried out. Pearson’s correlations were used for the correlation analyses, and Microsoft Excel 365 generated the heatmap visualization.

### 2.10. Statistical Analysis

All parameters were expressed as mean ± standard error (SE). Normal distributions were analyzed by the Kolmogorov–Smirnov test. Acetylcholine, choline, xanthurenic acid, sperm morphology, sperm viability, and acrosome integrity were analyzed using a one-way ANOVA followed by Duncan’s test. Other parameters were analyzed by the Kruskal–Wallis test and Mann–Whitney U test. The metabolomic profiles and sperm quality correlation were analyzed using Pearson’s correlations.

## 3. Results

### 3.1. Phytochemical Screening of WNPE Using Proton Nuclear Magnetic Resonance (^1^H-NMR)

The phytochemical screening of WNPE was carried out by performing a chemical shift and comparing the results with those of the specific library. The ^1^H-NMR spectra of the phytochemical profiles of WNPE were identified and analyzed. WNPE contained more phenolics and flavonoids than previously reported by LC-MS, including myricetin, apigenin, luteolin, ferulic acid, caffeic acid, ascorbic acid, genistein, chlorogenic acid, naringenin, and ellagic acid.

### 3.2. Analysis of Minerals in WNPE with AAS

The quantitation of 3 minerals (Mg, Zn, and Ca) in WNPE was expressed as mg/g of plant extract (Table 1).

### 3.3. Effect of White N. nucifera Petal Extract on Metabolomic Profile Determined by ^1^H-NMR

**Figure 1:**
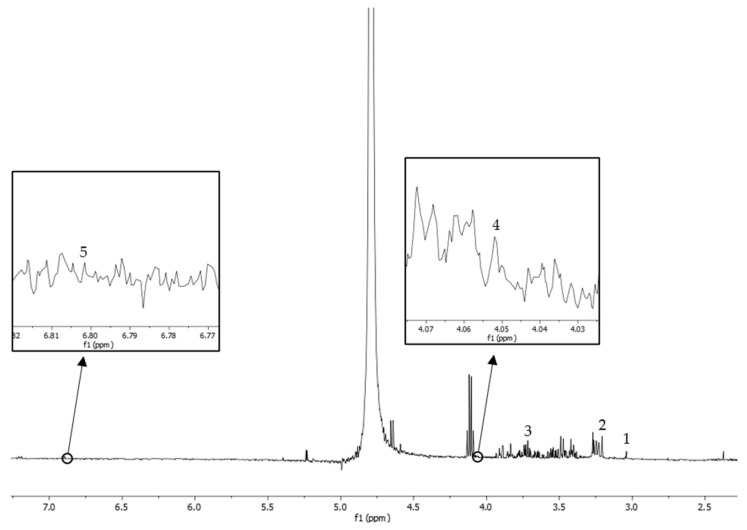
^1^H-NMR spectra from blood serum for peak identification: peak 1, creatine; peak 2, carnitine; peak 3, acetylcholine; peak 4, choline; peak 5, xanthurenic acid.

The ^1^H-NMR spectra of creatine (δ = 3.02), carnitine (3.21), acetylcholine (3.71), choline (δ = 4.05), and xanthurenic acid (δ = 6.80) were identified and analyzed (Figure 1). Creatine, carnitine, choline, and xanthurenic acid are the metabolomic profiles related to male reproductive antioxidant properties and function.

**Figure 2:**
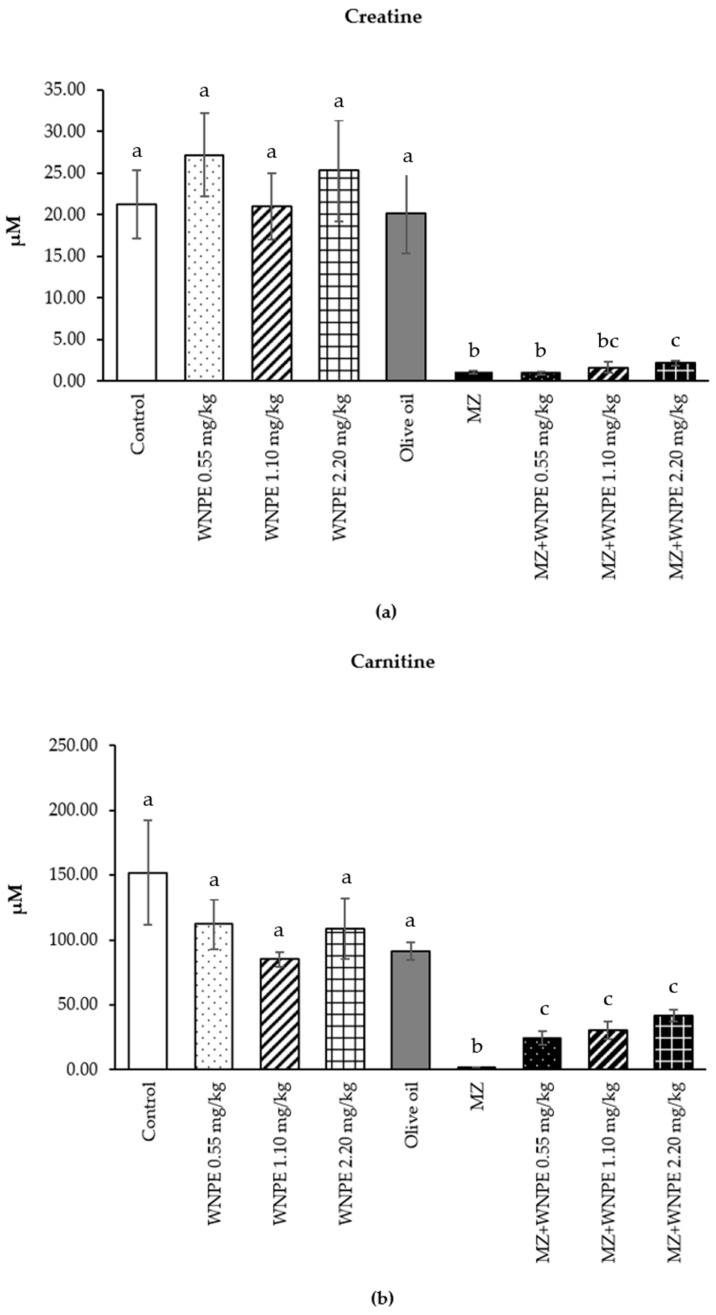

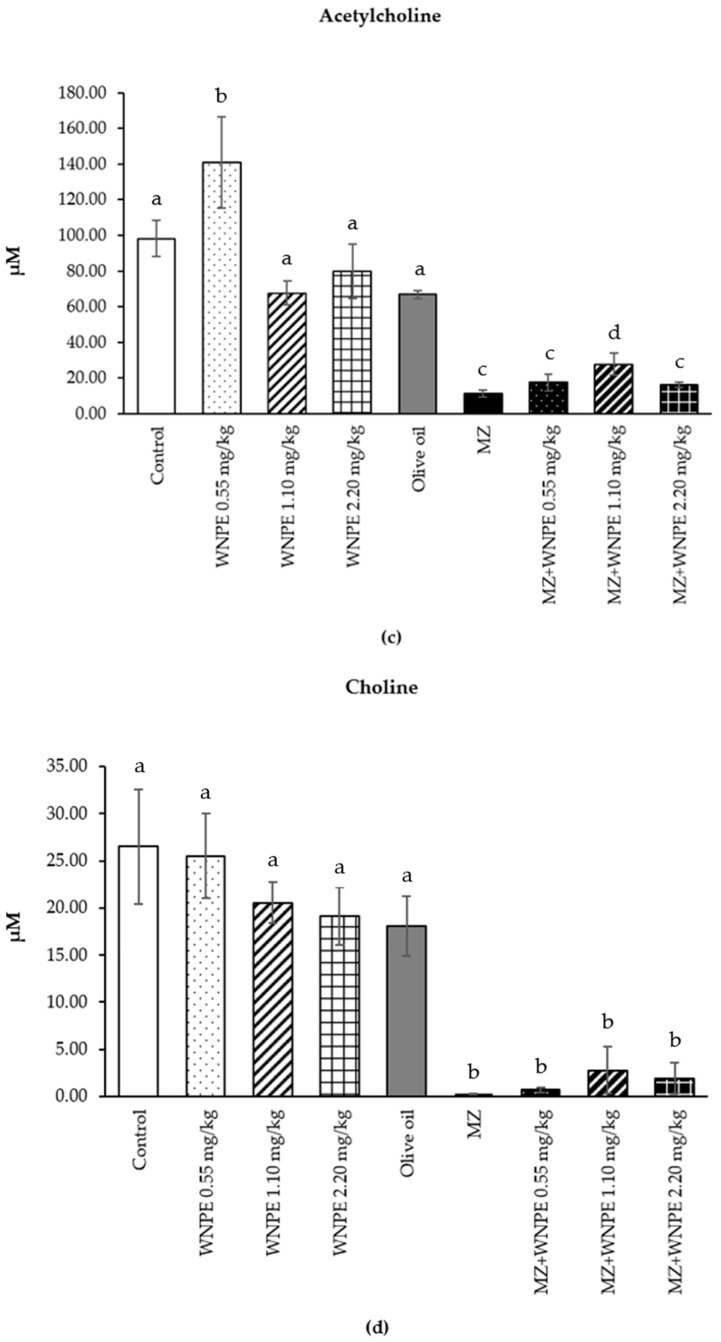

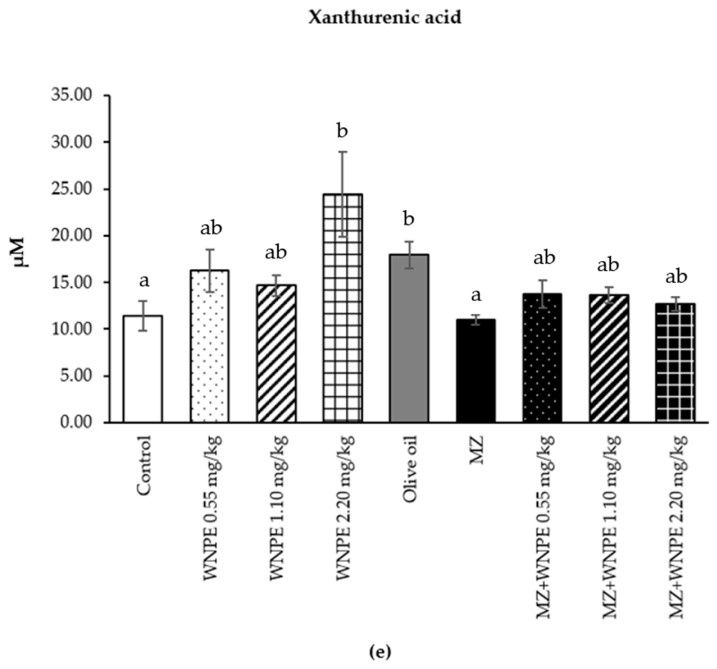
Creatine (**a**), carnitine (**b**), acetylcholine (**c**), choline (**d**), and xanthurenic acid (**e**) of mature male rats administered with different doses of white *N. nucifera* petals and MZ for 30 days (acetylcholine, choline, and xanthurenic acid were analyzed by one-way ANOVA followed by Duncan’s test, while other parameters were analyzed using the Kruskal–Wallis test followed by Mann–Whitney tests at *p* ≤ 0.05). ^a,b,c,d^ Different letters indicate significant differences between groups, while similar letters demonstrated no significant differences between groups. Data are presented as mean values ± SE (error bars).

The male rats receiving all doses of WNPE showed no significant difference compared to the control and vehicle control groups. However, the male rats receiving MZ had significantly lower creatine, carnitine, acetylcholine, and choline levels than the control and vehicle control groups. The male rats that were co-administered MZ and all doses of WNPE showed significantly improved carnitine levels compared to the MZ group. Creatine levels significantly improved in male rats receiving MZ and 2.20 mg/kg of WNPE compared to the MZ group. In addition, the male rats receiving 2.20 mg/kg of WNPE showed significantly higher levels of xanthurenic acid than the MZ and control groups, but they were similar to those of the vehicle control (Figure 2).

### 3.4. Sperm Quality

#### 3.4.1. Sperm Concentration and Sperm Motility

There was no significant alteration of sperm concentration in all treated groups when compared with the control, vehicle control, and MZ groups.

However, the percentages of motile and immotile sperm are presented (Table 2). The male rats receiving MZ showed a significant decrease in the percentage of progressive sperm motility. However, the percentage of progressive sperm motility significantly improved in the male rats receiving all doses of WNPE, those receiving olive oil (vehicle control), and those co-administered MZ and all doses of WNPE when compared with the MZ group. Moreover, the male rats receiving WNPE at a dose of 0.55 and 1.10 mg/kg and those co-administered MZ and WNPE at a dose of 0.55 mg/kg showed a significantly increase in the percentage of progressive sperm motility when compared with the control and vehicle control groups. In addition, the percentage of non-motile sperm was significantly increased in the rats receiving MZ compared with the control and vehicle control rats, while the rats receiving 1.10 and 2.20 mg/kg WNPE and those co-administered MZ and WNPE at the dose of 1.10 and 2.20 mg/kg showed improvement compared to those receiving MZ, which was similar to that of the control and vehicle control. Additionally, the rats receiving 0.55 mg/kg of WNPE and those co-administered MZ and WNPE at 0.55 mg/kg showed a significant decrease compared to the MZ, control, and vehicle control groups. The percentages of circle sperm were not significantly different among all experimental groups.

#### 3.4.2. Sperm Viability and Acrosome Integrity

All male rats receiving all doses of WNPE showed no significant difference compared to the control group in sperm viability and acrosome integrity. However, all male rats receiving all doses of WNPE showed significant differences compared to the MZ group in the number of viable sperm with intact acrosomes (Table 3).

The male rats receiving MZ showed a significant decrease in the number of viable sperm compared to the control and vehicle control, while there was a significant increase in dead sperm with detached acrosomes. The number of viable sperm with detached acrosomes significantly decreased in male rats that were co-administered MZ and all doses of WNPE when compared with the MZ group. Male rats co-administered MZ and 0.55 and 1.10 mg/kg of WNPE showed a significant increase in viable sperm with intact acrosomes compared to the MZ group. However, these groups had a significant decrease in dead sperm with detached acrosomes compared to the MZ group, which was similar to the control and vehicle control groups (Table 3).

#### 3.4.3. Sperm Morphology

All male rats receiving all doses of WNPE showed no significant difference compared to the control and vehicle control groups in the number of normal and abnormal sperm. However, all male rats receiving all doses of WNPE showed significant differences compared to the MZ group in the number of normal and abnormal sperm (Table 4).

**Figure 3:**
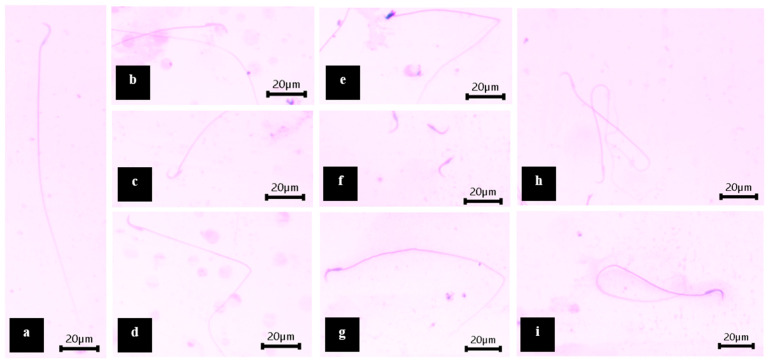
Morphological classification of rat sperm stained with trypan blue and counterstained with Giemsa. Normal sperm (**a**); sperm with different patterns of abnormal heads (**b**,**c**), including banana-shaped head (**b**) and microcephalus head (**c**); sperm with different patterns of abnormal heads and tails (**d**,**e**), including banana head with bent tail (**d**) and amorphous head with bent tail (**e**); and sperm with different patterns of abnormal tails (**f**–**i**), including no tail (**f**), bent tail (**g**), hairpin tail (**h**), and loop tail (**i**). Shown at a magnification of 400×.

The male rats receiving MZ showed a significant decrease in the number of normal sperm compared to the control and vehicle control, while there was a significant increase in the number of abnormal heads and abnormal heads and tails. The number of normal sperm significantly improved in male rats that were co-administered MZ and all doses of WNPE when compared with the MZ group. Male rats co-administered MZ and 0.55 and 1.10 mg/kg of WNPE showed a significant increase in viable sperm with intact acrosomes compared tp the MZ group. However, male rats co-administered MZ and 0.55 and 1.10 mg/kg of WNPE had a significant decrease in the number of abnormal heads and abnormal heads and tails compared to the MZ group, which is similar to the control and vehicle control groups (Table 4). Patterns of normal sperm and rat sperm with abnormal head, abnormal head and tail, and abnormal tail patterns are shown (Figure 3).

### 3.5. Correlation Analysis of Metabolomic Profiles and Sperm Quality

**Figure 4:**
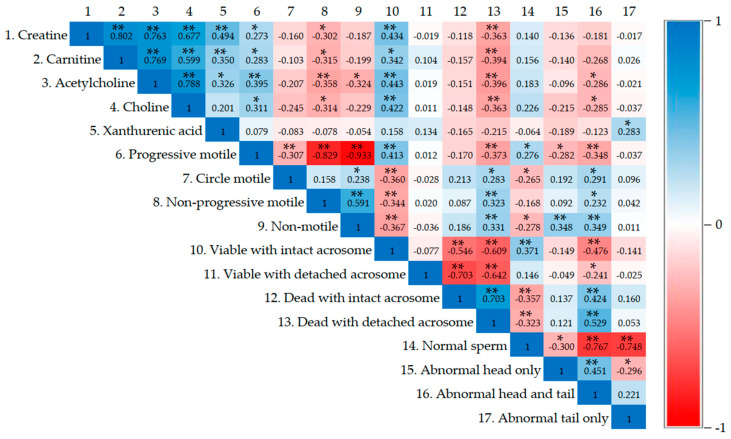
Heatmap visualization of Pearson’s correlations among metabolomic profiles and sperm quality present in male rats. Blue is a positive correlation, and red is a negative correlation. * Correlation is significant at 0.05. ** Correlation is significant at 0.01.

Correlation analysis was conducted between metabolomic profiles (creatine, carnitine, acetylcholine, choline, and xanthurenic acid) and sperm quality (Figure 4). This analysis revealed that creatine, carnitine, acetylcholine, and choline were positively correlated with progressive motility and viability with intact acrosomes, while they were negatively correlated with dead sperm with detached acrosomes. Moreover, progressive motility was positively correlated with viable sperm with intact acrosomes and a normal sperm morphology. However, progressive motility was negatively correlated with circle motility, non-progressive motility, non-motile sperm, dead sperm with detached acrosomes, and an abnormal sperm morphology.

## 4. Discussion

*Nelumbo nucifera* (*N. nucifera*) is a well-known plant and essential ingredient in traditional medicine and foods [33]. Various parts of N. nucifera are used as a medicine in Asia [35], especially in China, where it is one of the most economically important plants because of its various agricultural uses [36]. In our previous study, a phytochemical study of WNPE by LC-MS showed that it contained (+)-delta-tocopherol; kaempferitrin; ouabain; convallatoxin; salasodsalasodine; isorhamnetin-3-O-rutinoside; 20, 3, 3′4, 40-pentahydroxy-4′-glucosulchalcone; 4,8′-Bi((+)-epicatechin); and quercetin-3-O-arabinoglycoside [11]. The present study uses ^1^H-NMR for qualitative separation and detection of more phenolics and flavonoids than previously reported by LC-MS, including myricetin, apigenin, luteolin, ferulic acid, caffeic acid, ascorbic acid, genistein, chlorogenic acid, naringenin, and ellagic acid. Apigenin, myricetin, ferulic acid, ellagic acid, caffeic acid, genistein, and chlorogenic acid potentially increase sperm concentration, sperm motility, and sperm viability [44,45,46,47]. In addition, luteolin and naringin can prevent testicular injury [48,49]. Phenolics and flavonoids are common chemical substances in plants and have strong natural antioxidant activity, which is reported to increase the potential of free radical scavenging, the number of rapid progressive sperm, and the ability to preserve sperm [50]. Moreover, WNPE consists of essential minerals, especially Mg, Zn, and Ca, which have been reported to serve an essential function in spermatogenesis regarding sperm production, maturation, motility, and fertility [38]. In addition, in regard to NMR metabolomics, there is now a tool for the rapid and reproducible acquisition of metabolite profiles [23], allowing for the detailed examination of phytochemicals.

The male rats receiving MZ had significantly lower creatine, carnitine, acetylcholine, and choline levels in serum than the normal control, while those co-administered with MZ and WNPE showed significantly improved carnitine levels in all doses and significantly improved acetylcholine levels in the medium dose. Previous reports indicated that creatine is the main metabolite of the metabolomic profiles which is related to the male reproductive antioxidant properties and function [26,51,52]. Creatine, the most often used ergogenic supplement, supports the preservation of ATP levels during periods of high energy demand and has antioxidant properties [51]. The testis is one of the body’s primary sources of creatine [53]. It is thought to play an important role in both male and female germ cell development, but its exact role in the male reproductive function is not known [54]. Acetylcholine (Ach) is a neurotransmitter that stimulates calcium (Ca^2+^) influx, activating a Ca^2+^-dependent pathway that includes activation of adenylyl cyclase activity and increased cyclic AMP (cAMP) levels, resulting in sperm motility. Furthermore, acetylcholine enzymes (AChEs) influence ACh to hydrolyze into acetic acid and choline. Choline is transported up to the neuron and resynthesized into new ACh [55]. MZ is the most commercially available of all ethylene bis dithiocarbamates (EBDCs) and is widely used for fungal control in agriculture and industry [2]. MZ toxicity occurs due to AChE inhibition, which blocks the hydrolysis of ACh into choline, resulting in a reduction in the amount of choline available for resynthesizing into new ACh and a decline in Ach [56]. In addition, MZ contains heavy metals that induce oxidative stress and male reproductive dysfunction [2,11]. In our previous study, oxidative stress molecular markers, including LPO markers, AOPP markers, and AGEs markers from serum, were increased in male rats receiving MZ. At the same time, the co-administering of WNPE and MZ caused a significant decrease in these markers compared to the MZ group. In addition, the MZ group saw a significant decrease in glutathione levels compared to the control group, whereas the co-administering of WNPE and MZ saw an improvement in these levels compared to the MZ group [6]. Oxidative stress causes cellular damage [14,15], which reduces spermatogenesis, the physiological potential for fertilization, and male fertility [16,17,18]. However, glutathione is an antioxidant that prevents reproductive toxicity in diabetic mice via improved spermatogenesis and sperm quality [57]. MZ may decrease metabolomic profiles related to antioxidant activity and male reproductive function, while WNPE has the potential to improve the metabolomic profiles and antioxidant activity despite MZ exposure.

The sperm motility assay was modified from the conventional technique used for the study of rat sperm motility [43]. This method is available and necessary for collecting data quickly and in real-time, it can be used to collect data from many aspects at the same time, and it correlates with the sperm motility analysis system [58,59]. Sperm motility and viability, acrosome integrity, and normal sperm morphology were identified to determine sperm quality. The male rats that were poisoned with MZ showed a decreasing value in all areas. These results were similar to those of our in vitro study, which found that MZ had a toxic effect on bull sperm, resulting in reduced sperm function, motility, membrane integrity, and acrosome activity [11]. Moreover, male rats administered 800 mg/mL of MZ may pass on its effects, including reduced sperm motility and viability, to their descendants [60]. Consequently, applying WNPE in combination with MZ in animal models is interesting. Sperm quality, especially acrosome integrity, is essential to oocyte penetration and fertilization [61]. MZ consists of heavy metals, and its metabolization creates ETU, which can cause free radicals and inhibit AChE ability, leading to overstimulation of the Ach receptor and resulting in decreased choline and ACh levels, which reduce Ca^2+^ influx and sperm motility [56]. Moreover, carbamates from MZ demonstrated toxicity via knockdown of the muscarinic ACh (mACh) receptor, resulting in disrupted spermatogenesis and spermiogenesis that affected sperm quality [56,62]. The results showed that MZ adversely affects the male reproductive system, directly affecting sperm in vitro [11] and the body’s metabolism in vivo. However, this study found that all doses of WNPE had a positive effect on the male rats and enhanced progressive sperm motility, normal sperm morphology, sperm viability, and acrosome integrity, especially at the dose of 0.55 mg/kg which showed enhanced progressive sperm motility compared to the MZ, control, and vehicle control groups. This result was similar to that of the WNAE treatment in the cattle sperm with induced toxicity by MZ, which demonstrated a decrease in immotile sperm in all doses of WNAE when compared with the MZ group and found that a low dose of WNAE led to a significant decline in non-motile sperm when compared to the control [11]. The results indicated that white *N. nucifera* petal extract could potentially prevent MZ toxicity both in vitro and in vivo. Moreover, the result was consistent with the previous report that gallic acid as a phenolic compound had the potential to increase rapid progressive sperm in mice [50]. Similarly, the *Bombax ceiba* stamen extract that contains gallic acid had the potential to increase progressive sperm motility, sperm viability, and normal sperm morphology [63]. The WNPE may contain various bioactive compounds that might directly affect repro-protection in male rats induced with MZ, which is similar to the findings from the in vitro study in cattle sperm induced with MZ. Sperm motility is the cause of male infertility, which is the final step of spermiogenesis where the mitochondria are arranged in the middle part of the sperm tail [64]. The study of sperm quality, such as sperm motility, sperm viability, and sperm head morphology, can predict nucleus instability and insufficient sperm protein that determines the rate of success in male fertility [65]. However, WNPE contained essential phytochemical compounds including phenols, flavonoids, and alkaloids [11,32]. Flavonoids and alkaloids had benefits for male reproductive function and sperm preservation [66,67,68]. Moreover, bioactive compounds such as phenolics and flavonoids have strong natural antioxidants that scavenge free radicals and promote healthy sperm [67,68]. It is possible that the synergism of the phytochemicals was beneficial for the male reproductive system via decreases in oxidative stress and MZ toxicity, which enhance ACh synthesis and prevent ACh receptor knockdown and which promote spermiogenesis and healthy sperm.

The correlation between the metabolomic profiles and sperm quality revealed that creatine, carnitine, acetylcholine, and choline were positively correlated with progressive motility, or the number of viable sperm with intact acrosomes. Consistently, it has been previously reported that creatine can be used to support spermatogenesis and sperm motility, and the testis is the primary source of creatine [53]. ACh can hydrolyze into acetic acid and choline, and choline causes an increase in intracellular calcium in sperm, resulting in a contribution to sperm motility, which is related to the success of fertility [52]. Additionally, progressive motility was positively correlated with viable sperm with intact acrosomes and normal sperm morphology. The normal sperm morphology and viability of sperm cause progressive sperm motility, resulting in the increased rate of success in male fertility [38]. Therefore, the secondary metabolites of WNPE, especially in low doses which have a spectrum of bioactive compounds, affect sperm quality.

MZ had a toxic effect on the male reproductive system via inhibited AChEs, and decreased sperm motility, sperm viability, and normal sperm morphology. However, the WNPE contained plenty of phytochemical compounds and demonstrated enhanced antioxidant properties, such as raised carnitine levels in all doses and raised acetylcholine levels in the medium dose. This suggests that the WNPE improved the sperm quality, including the sperm motility, sperm viability, and normal sperm morphology, of male rats that were induced with MZ toxicity as the WNPE protected against oxidative stress from MZ, resulting in higher male reproductive function potential. As a result, the antioxidative phytochemicals of WNPE, especially at the dose of 0.55 mg/kg, operate as antioxidants, protecting the male reproductive system against oxidative stress and toxicity caused by MZ exposure.

## 5. Conclusions

In conclusion, MZ had a toxic effect on the male reproductive system via inhibited AChEs and the knockdown of ACh receptors, leading to decreased sperm quality. WNPE contains plenty of phytochemical compounds, making it a strong natural antioxidant that has the potential to protect against oxidative stress and toxicity in male rats poisoned with MZ. The WNPE enhances sperm quality, which is related to male rat reproductive function. It is suggested that WNPE should be considered as an alternative dietary supplement to protect against MZ toxicity.

## Figures and Tables

**Table 1:** The mean value of Mg, Zn, and Ca in WNPE (mean ± SE.).

Group	Mineral Composition (mg/g of Plant Extract)		
	Mg	Zn	Ca
WNPE	14.83 ± 0.04	0.11 ± 0.01	5.20 ± 0.05

**Table 2:** Percentage of motile and non-motile sperm of mature male rats treated with white *N. nucifera* petal extract compared with the control and MZ groups.

Group	Percentages of Motile Sperm			Percentages of Non-Motile Sperm
	Progressive	Circle	Non-Progressive	
Control (*n* = 8)	61.31 ± 1.82 ^a^	2.06 ± 0.27	11.13 ± 1.50 ^ab^	25.50 ± 1.78 ^acd^
0.55 mg/kg (*n* = 8)	79.50 ± 3.91 ^b^	0.94 ± 0.11	8.63 ± 1.95 ^a^	10.94 ± 2.28 ^b^
1.10 mg/kg (*n* = 8)	68.00 ± 1.39 ^c^	1.56 ± 0.53	9.38 ± 0.99 ^a^	21.06 ± 0.85 ^d^
2.20 mg/kg (*n* = 8)	64.81 ± 3.92 ^ac^	1.31 ± 0.33	12.56 ± 2.03 ^ab^	21.31 ± 2.61 ^ad^
Olive oil (*n* = 8)	60.38 ± 2.58 ^a^	2.75 ± 0.69	11.81 ± 1.24 ^ab^	25.06 ± 1.33 ^ad^
MZ (*n* = 8)	39.50 ± 8.07 ^d^	2.44 ± 0.64	19.06 ± 3.40 ^b^	39.69 ± 5.00 ^e^
MZ + 0.55 mg/kg (*n* = 8)	74.94 ± 4.46 ^bc^	1.19 ± 0.23	12.31 ± 3.33 ^ab^	11.56 ± 1.48 ^bc^
MZ + 1.10 mg/kg (*n* = 8)	57.88 ± 2.79 ^a^	2.06 ± 0.41	15.31 ± 1.85 ^b^	25.38 ± 1.01 ^a^
MZ + 2.20 mg/kg (*n* = 8)	58.50 ± 3.89 ^a^	2.06 ± 0.48	15.13 ± 2.29 ^b^	24.31 ± 2.06 ^ad^

All parameters were analyzed using the Kruskal–Wallis test followed by Mann–Whitney tests at *p* ≤ 0.05. All values are presented as mean ± SE. ^a,b,c,d,e^ Different letters indicate significant differences between groups in column data, while similar letters demonstrate no significant differences between groups.

**Table 3:** Numbers indicating sperm viability (viable and dead) and acrosome integrity (intact and detached acrosomes) in mature male rats treated with white *N*. *nucifera* petals, as well as for the control and MZ groups.

Group	Number of Viable Sperm		Number of Dead Sperm	
	Intact	Detached	Intact	Detached
Control (*n* = 8)	33.88 ± 4.74 ^a^	45.75 ± 2.80 ^ab^	12.88 ± 3.58 ^a^	7.50 ± 2.08 ^a^
0.55 mg/kg (*n* = 8)	41.00 ± 6.15 ^a^	41.75 ± 6.15 ^ab^	10.88 ± 3.49 ^a^	6.38 ± 2.24 ^a^
1.10 mg/kg (*n* = 8)	38.25 ± 6.24 ^a^	33.13 ± 5.60 ^bc^	17.50 ± 4.89 ^a^	11.13 ± 4.20 ^ab^
2.20 mg/kg (*n* = 8)	35.75 ± 7.07 ^a^	36.75 ± 6.95 ^bc^	17.13 ± 6.52 ^a^	10.50 ± 2.96 ^ab^
Olive oil (*n* = 8)	28.63 ± 5.10 ^ab^	47.38 ± 5.41 ^ab^	14.88 ± 4.55 ^a^	9.13 ± 2.14 ^a^
MZ (*n* = 8)	14.88 ± 4.78 ^b^	20.88 ± 6.52 ^c^	32.63 ± 4.95 ^b^	31.63 ± 7.26 ^c^
MZ + 0.55 mg/kg (*n* = 8)	33.75 ± 6.77 ^a^	43.38 ± 8.07 ^ab^	11.38 ± 5.54 ^a^	11.50 ± 6.00 ^ab^
MZ + 1.10 mg/kg (*n* = 8)	25.75 ± 3.96 ^ab^	59.75 ± 5.85 ^a^	6.00 ± 2.07 ^a^	8.50 ± 1.90 ^a^
MZ + 2.20 mg/kg (*n* = 8)	15.38 ± 3.41 ^b^	50.25 ± 6.96 ^ab^	12.25 ± 2.21 ^a^	22.13 ± 3.68 ^bc^

All parameters were analyzed using one-way ANOVA followed by Duncan’s test at *p* ≤ 0.05. All values are presented as mean ± SE. ^a,b,c^ Different letters indicate significant differences between groups in column data, while similar letters demonstrate no significant differences between groups.

**Table 4:** Numbers of the normal and abnormal sperm of mature male rats treated with white *N. nucifera* petals, as well as for the control and MZ groups.

Group	Number of Normal Sperm	Number of Abnormal Sperm		
		Head Only	Head and Tail	Tail Only
Control (*n* = 8)	57.50 ± 4.72 ^a^	5.88 ± 0.74 ^a^	7.50 ± 1.70 ^a^	29.13 ± 3.51
0.55 mg/kg (*n* = 8)	58.38 ± 4.72 ^a^	6.88 ± 1.60 ^a^	7.63 ± 2.14 ^a^	27.13 ± 4.62
1.10 mg/kg (*n* = 8)	55.00 ± 5.89 ^a^	7.00 ± 1.02 ^a^	7.75 ± 2.06 ^a^	30.25 ± 5.34
2.20 mg/kg (*n* = 8)	49.75 ± 5.06 ^a^	6.38 ± 1.49 ^a^	8.13 ± 2.36 ^a^	35.75 ± 4.62
Olive oil (*n* = 8)	52.25 ± 5.39 ^a^	8.63 ± 1.19 ^a^	9.00 ± 1.71 ^a^	30.13 ± 4.17
MZ (*n* = 8)	34.63 ± 3.90 ^b^	13.50 ± 1.99 ^b^	21.25 ± 3.00 ^b^	30.63 ± 2.58
MZ + 0.55 mg/kg (*n* = 8)	57.25 ± 3.66 ^a^	5.63 ± 1.28 ^a^	7.50 ± 1.38 ^a^	29.63 ± 3.05
MZ + 1.10 mg/kg (*n* = 8)	62.13 ± 3.21 ^a^	5.38 ± 0.98 ^a^	7.00 ± 1.09 ^a^	25.50 ± 2.68
MZ + 2.20 mg/kg (*n* = 8)	55.25 ± 4.38 ^a^	5.50 ± 1.72 ^a^	8.88 ± 1.47 ^a^	30.38 ± 3.17

All parameters were analyzed using one-way ANOVA followed by Duncan’s test at *p* ≤ 0.05. All values are presented as mean ± SE. ^a,b^ Different letters indicate significant differences between groups in column data, while similar letters demonstrate no significant differences between groups.

## Data Availability

The authors state that the data supporting the results of this study are available in this article and Supplementary Materials.

## Supplementary Materials

The following supporting information can be downloaded at: https://www.mdpi.com/article/10.3390/life15010006/s1.
