# Supplementary material for: The Potential Effect of Bualuang (White Nelumbo nucifera Gaertn.) Extract on Sperm Quality and Metabolomic Profiles in Mancozeb-Induced Oxidative Stress in Male Rats
Source: Life (Basel). 2024 Dec 24;15(1):6. doi: 10.3390/life15010006 (PMC11767100; doi:10.3390/life15010006)
Supplement: Supplementary file 1 [file life-15-00006-s001.zip › life-3351818-supplementary.pdf]

Supplement data

Phytochemical Screening of WNPE Using Proton Nuclear Magnetic Resonance (^{1}H-NMR)

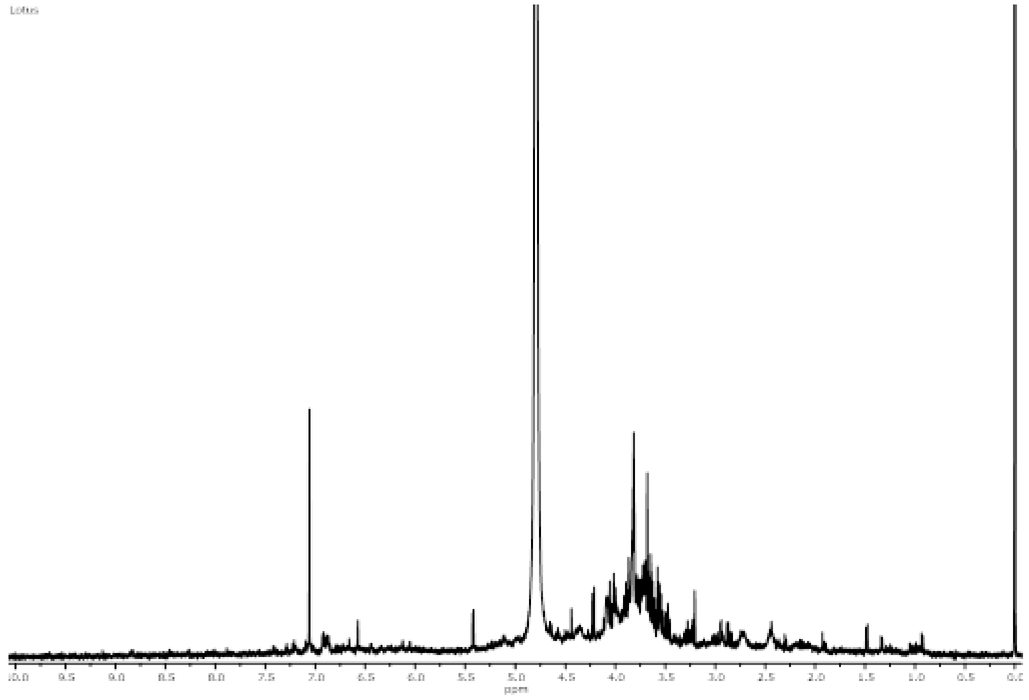

Figure S1. ^{1}H-NMR spectra from white *N. nuceifera* petal extract dissolved in deuterium oxide (D_2O) and trimethylsilyl propanoic acid (TSP), which was added as an internal reference for chemical shift (0 ppm).

Table S1. ^{1}H-NMR profile of white *N. nuceifera* petal extract.

No.	Compounds	HMDB	Identifications
1	quercetin	0005794	6.20(s), 6.42(s), 6.89(d;J=9.6), 7.55(dd;J=7.5,0.6), 7.68(s)
2	Myricetin	0002755	6.18(s), 6.37(s), 7.34(s)
3	Kaempferol	0005801	6.18(d;J=0.5), 6.39(d;J=0.9), 6.90(d;J=10.0), 8.08(d;J=9.9)
4	isorhamnetin	0002655	3.68(s), 6.23(d;J=0.4), 6.43(d;J=0.8), 6.97(d;J=9.2), 8.25(d;J=9.6), 8.29(s)
5	Apigenin	0002124	6.13(d;J=0.8), 6.43(d;J=0.8), 6.74(s), 6.92(d;J=6.9), 7.72(d;J=10.4)
6	Myricetin 3′ glucoside	0034359	2.86(dd;J=17.0,7.7), 3.49(t;J=4.4), 3.75(dd;J=15.8,3.3), 4.97(d;J=0.6), 6.12(s), 6.71(s)
7	Kaempferol 3-sophorotrioside	0032008	3.49(m), 3.63(dd;J=9.6,7.1), 3.75(dd;J=8.0, 4.8), 3.81(m), 3.84(dd;J=8.8, 4.0), 6.12(s), 6.87(s), 6.87(dd;J=7.9, 1.1), 6.93(d;J=5.0)

8	Isorhamnetin 3- bete-laminarbioside	0041383	2.86(s), 3.49(s), 3.63(s), 3.75(s), 3.81(s), 3.85(s), 4.12(s), 5.18(s), 6.12(s), 6.71(s), 6.81(s), 6.93(s)
9	Isorhamnetin 3- beta-D-glucoside	0302682	3.21(m), 3.46(t;J=22.7), 3.51(dd;J=13.5,4.9), 3.82(t;J=6.8), 3.86(s), 4.04(d;J=5.0), 5.31(d;J=3.8), 6.23(d;J=0.4), 6.43(d;J=0.8), 6.97(d;J=5.0), 7.55(d;J=4.4), 7.91(s)
10	Calendoflavoside	0037745	1.13(d;J=5.7), 3.49(m), 3.63(t;J=2.8), 3.73(dd;J=10.0,4.4), 3.75(dd;J=8.0,4.8), 3.81(s), 4.12(m), 5.18(t;J=3.7), 6.09(d;J=0.5), 6.71(d;J=0.8), 6.87(d;J=9.5), 6.93(d;J=10.4), 7.14(d;J=0.4)
11	Diosmin	0029548	1.13(d;J=5.6), 3.49(s), 3.85(m), 3.87(s), 4.12(d;J=4.7), 6.12(d;J=1.6), 6.51(d;J=0.9), 6.71(s), 6.94(d;J=10.7), 7.04(s), 7.06(d;J=9.4)
12	Myricetin 3-galactoside	0034358	3.49(t;J=6.0), 3.75(dd;J=6.3,3.0), 3.84(dd;J=8.8,4.0), 4.12(t;J=9.0), 4.26(m), 4.37(dd;J=3.7, 2.9), 5.63(d;J=5.2), 6.12(s), 6.71(s)
13	Myricetin 3-neohesperidoside	0038287	1.38(d;J=5.9), 3.42(dd;J=7.6, 0.9), 3.49(dd;J=11.0, 8.4), 3.63(s), 3.75(d;J=8.7), 3.87(m), 4.12(s), 5.18(s), 6.12(s), 6.71(s)
14	Myricetin 3,3′-digalactoside	0037850	3.49(d;J=1.5), 3.75(d;J=8.7), 3.85(t;J=5.5), 4.03(dd;J=9.6, 1.9), 4.37(m), 5.63(d;J=5.2), 6.12 (s), 6.71(s), 7.29(s)
15	Myricetin 3- glucoside	0303631	3.21(t;J=10.3), 3.46(d;J=9.6), 3.51(dd;J=7.9, 0.9), 3.82(s), 4.04(d;J=5.0), 5.30(d;J=9.4), 6.23(d;J=0.5), 6.43(d;J=0.7), 7.60(s)
16	Myricetin 3-robinobioside	0040861	1.38(d;J=5.9), 3.42(t;J=11.0), 3.63(t;J=21.1), 3.68(t;J=6.0), 3.70(dd;J=20.5, 8.1), 3.80(s), 4.02(m), 4.25(dd;J=20.9, 9.6), 4.37(dd;J=10.5, 0.8), 5.18(m), 5.63(s), 6.09(d;J=0.5), 6.71(s), 7.29(s)
17	luteolin	0005800	3.31(s), 6.23(s), 6.46(s), 6.56(s), 6.92(d;J=1.4), 7.40(s)
18	Quercetin 4′-glucoside	0037932	3.49(ddd;J=4.7,3.0,1.6), 3.56(m), 3.75(d;J=6.3), 3.85(d;J=3.5), 6.09(d;J=0.5), 6.12(d;J=1.6), 6.51(d;J=9.6), 6.93(d;J=11.2), 7.04(s)
19	Rutin	0003249	1.389(d;J=5.1), 3.49(t;J=3.0), 3.70(dd;J=4.0, 1.7), 3.71(ddd;J=11.1, 6.5, 2.2), 3.84(dd;J= 9.0, 6.8), 4.05(ddd;J=15.8,10.7, 7.4), 4.12(t;J=8.4), 4.37(t;J=4.2),

			4.97(s),5.18(d;J=5.1), 6.09(s), 6.71(s), 6.93(d;J=6.6), 7.05(d;J=4.0)
20	Diosmetin 8-C-c2''-rhamnosyl glucoside	0037448	1.13(d;J=5.6), 3.49(dd;J=9.0, 1.4), 3.63(dd;J=10.6, 10.1), 3.75(d;J=6.0), 3.85(d;J=3.0), 3.87(s), 4.12(t;J=9.0), 5.18(t;J=9.8), 6.09(s), 6.71(s), 6.87(d;J=10.6), 7.06(d;J=9.4)
21	Diosmetin 7-neohesperidoside	0039856	1.13(d;J=5.6), 2.86(d;J=1.4), 3.49(dd;J=9.0, 1.4), 3.63(dd;J=10.6, 10.1), 3.75(d;J=6.0), 3.85(dd;J=7.0, 3.5), 3.87(s), 4.12(dt;J=13.8, 8.8), 5.18(d;J=5.1), 6.12(d;J=1.6), 6.51(s), 6.71(s), 7.05(d;J=8.6), 7.06(d;J=9.4)
22	Apigenin 7-[galactosyl-1(1→4) mannoside	0037852	2.86(dd;J=17.0, 7.7), 3.49(dd;J=10.9, 1.5), 3.63(dd;J=11.8,0.3), 3.75(dd;J=8.0, 4.8), 4.12(t;J=8.8), 5.18(s), 6.12(d;J=0.4), 6.51(s), 6.71(d;J=0.8), 6.93(d;J=5.6), 7.06(d;J=9.9)
23	Orientin	0030614	3.49(dd;J=11.1, 4.1), 3.75(d;J=9.3), 4.12(dd;J=7.0, 4.0), 4.37(d;J=0.5), 4.95(s), 6.12(s), 6.71(s), 6.78(d;J=5.9), 6.93(d;J=5.6), 7.04(d;J=1.2)
24	Scolymoside	0005799	1.38(d;J=6.0), 3.42(d;J=10.3), 3.63(t;J=2.8), 3.75(dd;J=9.6, 3.3), 3.81(m), 3.84(dd;J=10.1, 5.2), 4.03(d;J=0.9), 4.05(d;J=1.3),4.25(dd;J=20.4, 9.4), 4.37(dd;J=3.4, 0.7), 5.18(s), 5.46(d;J=5.4), 6.51(s), 6.71(s), 6.88(d;J=5.4), 6.93(d;J=8.9)
25	4-hydroxybenzoic acid	0000500	6.91(d;J=0.5), 7.80(dd;J=2.6,1.1), 7.80(dd;J=2.6, 1.1)
26	Chlorogenic acid	0003164	2.01(dd;J=6.8,2.3), 2.03(dd;J=8.1,3.0), 2.13(d;J=6.9), 2.25(t;J=3.3), 3.86(dd;J=10.1,3.0), 4.26(t;J=3.9), 5.32(ddd;J=4.9,3.1,0.7), 6.51(d;J=2.7), 6.78(d;J=1.9), 7.05(dd;J=1.5,0.8), 7.06(d;J=0.9), 7.53(d;J=1.8)
27	Ferulic acid	0000954	3.89(s), 6.37(d;J=2.1), 6.91(d;J=2.2), 7.11(dd;J=2.9,1.3), 7.22(d;J=1.1), 7.31(d;J=7.4)
28	Vanillic acid	0000484	3.90(s), 6.94(d;J=2.3), 7.45(t;J=6.0), 7.52(s)
29	Caffeic acid	0001964	6.29(d;J=1.4), 6.80(d;J=3.6), 6.96(dd;J=2.8,2.0), 7.03(d;J=1.9), 7.55(d;J=1.5)
30	Trans-cinnamic acid	0000930	6.52(d;J=8.8), 7.43(m), 7.62(t;J=6.9)
	4-hydroxycinnamic acid	0002035	6.50(d;J=16.6), 6.79(d;J=2.2), 7.39(d;J=5.3), 7.72(d;J=2.2)

	Ascorbic acid	0000044	3.44(d;J=5.7), 3.73(dd;J=11.3,3.5), 4.72(d;J=5.2)
31	3-Hydroxy-beta-ionol 3-[glucosyl-(1->6)-glucoside]	0037527	1.12(s), 1.30(d;J=5.5), 1.75(s), 1.86(m), 2.58(m), 3.49(t;J=14.2), 3.63(dd;J=11.9,7.1), 3.70(m), 3.75(dd;J=12.9,3.9), 3.85(d;J=2.5), 3.91(t;J=4.2), 4.05(dd;J=8.6,5.2), 4.12(d;J=7.4), 5.18(d;J=3.5), 6.65(dd;J=2.4,1.2), 7.28(d;J=2.6)
32	Cyanidin	0002708	6.12(s), 6.51(s), 6.78(d;J=7.9), 6.93(dd;J=1.8,0.8), 8.07(d;J=0.4)
33	Harman	0035196	3.35(s), 7.12(d;J=3.3), 7.27(t;J=3.8), 7.42(t;J=6.8), 7.72 (d;J=1.3), 8.19(d;J=1.6), 8.28(d;J=4.8)
34	genistein	0003217	6.38(s),6.39(s), 6.95(d;J=3.9), 7.30(d;J=1.6), 7.62(s)
35	4-hydroxybenzoic acid	0000500	6.91(d;J=0.5), 7.80(dd;J=2.6,1.1), 7.80(dd;J=2.6, 1.1)
36	Chlorogenic acid	0003164	2.01(dd;J=6.8,2.3), 2.03(dd;J=8.1,3.0), 2.13(d;J=6.9), 2.25(t;J=3.3), 3.86(dd;J=10.1,3.0), 4.26(t;J=3.9), 5.32(ddd;J=4.9,3.1,0.7), 6.51(d;J=2.7), 6.78(d;J=1.9), 7.05(dd;J=1.5,0.8), 7.06(d;J=0.9), 7.53(d;J=1.8)
37	Capric acid	0000511	0.88(t;J=1.1), 1.26(m), 1.27(m), 1.28(m), 1.56(dt;J=14.2,8.0), 2.30(t;J=2.9)
38	Gallic acid	0005807	6.93(d;J=0.4), 7.29(d;J=0.4)
39	Isoferulic acid	0000955	3.89(s), 6.36(d;J=15.2), 7.04(d;J=10.1), 7.14(t;J=9.6), 7.29(d;J=15.6)
40	isoquercetin	0037362	3.75(d;J= 20.0,5.2), 3.84(dd;J=10.2,2.9), 3.87(dd;J= 8.8,5.0), 6.09(s), 6.12(d;J=0.6), 6.71(s), 6.88(d;J= 11.8), 6.93(d;J=9.6)
41	Piceid 3-sulfate	0240553	3.49(t;J=10.0), 3.56(dt;J=18.2,7.5), 3.72(d;J=5.9), 3.90(dd;J= 8.4, 3.7), 4.12(t;J=9.4), 4.62(d;J= 9.4), 6.12(s), 6.51(s), 6.78(dd;J=9.9,0.5), 6.88(dd;J=5.4,0.9), 7.06(d;J=5.4), 7.29(d;J=15.6), 7.50(dd;J= 7.5,1.3) 7.61(d;J=12.6)
42	naringenin	0002670	3.02(d;J=7.1), 551(dt;J=31.0,12.0), 6.13(s), 6.51(s), 6.81 (d;J=9.6)
43	kaempferol	0005801	6.18(d;J=0.6), 6.39(d;J=0.7), 6.90(d;J=9.3), 8.08(d;J=12.1)

44	Ellagic acid	0002899	7.46(s)
45	catechin	0002780	2.50(dd;J=15.7,6.6), 2.84(s), 3.97(ddd;J= 31.8,12.5,4.1), 4.56(d;J=7.9), 5.85(s), 5.92(s), 6.71(dd;J=11.3,10.6), 6.76(d;J=11.6), 6.83(d;J=0.8)
46	genistein	0003217	6.38(s), 6.39(s), 695(d;J=8.7), 7.30(d;J=8.0), 7.62(s)
47	Gentisic acid	0000152	6.81(dd;J=0.7,0.8), 6.84(d;J=7.5), 7.42(s)
48	Gentisuric acid	0059999	3.69(s), 6.88(s), 6.93(d;J=4.7)
49	Isovanillic acid	0060003	3.85(t;J=11.3), 3.97(d;J=12.3), 6.81(d;J=5.5), 7.25(d;J=0.4), 7.69(dd;J=4.0,1.0)
50	vanillin	0012308	3.83(s), 6.78(d;J=8.4), 7.29(d;J=0.4), 7.41(dd;J= 3.6, 0.5), 9.46(s)
51	Methyl-4-methylpentanoate	0036238	0.88(m), 0.92(dd;J=7.6,2.7), 1.40(dd;J=6.8,3.3), 2.08(m), 2.53(t;J=9.8), 3.70(s)
